# Inequities in Canadian maternal-child healthcare are perpetuating the intergenerational effects of colonization for indigenous women and children

**DOI:** 10.3389/fgwh.2025.1513145

**Published:** 2025-06-04

**Authors:** Andrea M. Weckman, Patricia Farrugia

**Affiliations:** ^1^ Michael G. DeGroote School of Medicine, McMaster University, Hamilton, ON, Canada; ^2^ Department of Orthopedic Surgery, McMaster University, Hamilton, ON, Canada

**Keywords:** indigenous, maternal-child health, decolonization, reproductive justice, intergenerational

## Abstract

Universal, publicly funded healthcare has long been a point of pride for Canada, despite decades of data contradicting its universality and accessibility. Inequities in access to and provision of healthcare services are particularly evident in the direct comparison of health outcomes between Indigenous (First Nations, Inuit, and Métis) and non-Indigenous populations in Canada. Globally, there are data to support similar disparities in maternal-child health for Indigenous populations around the world. Here, we describe how these inequities uniquely impact people at the intersection of multiple vulnerabilities—Indigenous pregnant women and their children. Indigenous pregnant women in Canada are far more likely to have experienced harmful *in utero* exposures, inadequate antenatal care, and adverse birth outcomes than non-Indigenous pregnant women. These inequities in maternal-child health may be contributing to biological processes (e.g., epigenetic reprogramming) with intergenerational consequences for chronic disease risk in Indigenous populations. We highlight how the current state of maternal-child health for Indigenous women in Canada is likely perpetuating the multigenerational cycle of oppression triggered by the process of colonization. Finally, we outline current efforts to achieve reproductive justice, decolonize maternal-child health in Canada, and reclaim childbirth by Indigenous communities and their allies. We recognize the strength and resilience of Indigenous women in Canada to resist the persistence of colonial ideals in birthing rights and practices.

## Introduction

**Figure 1:**
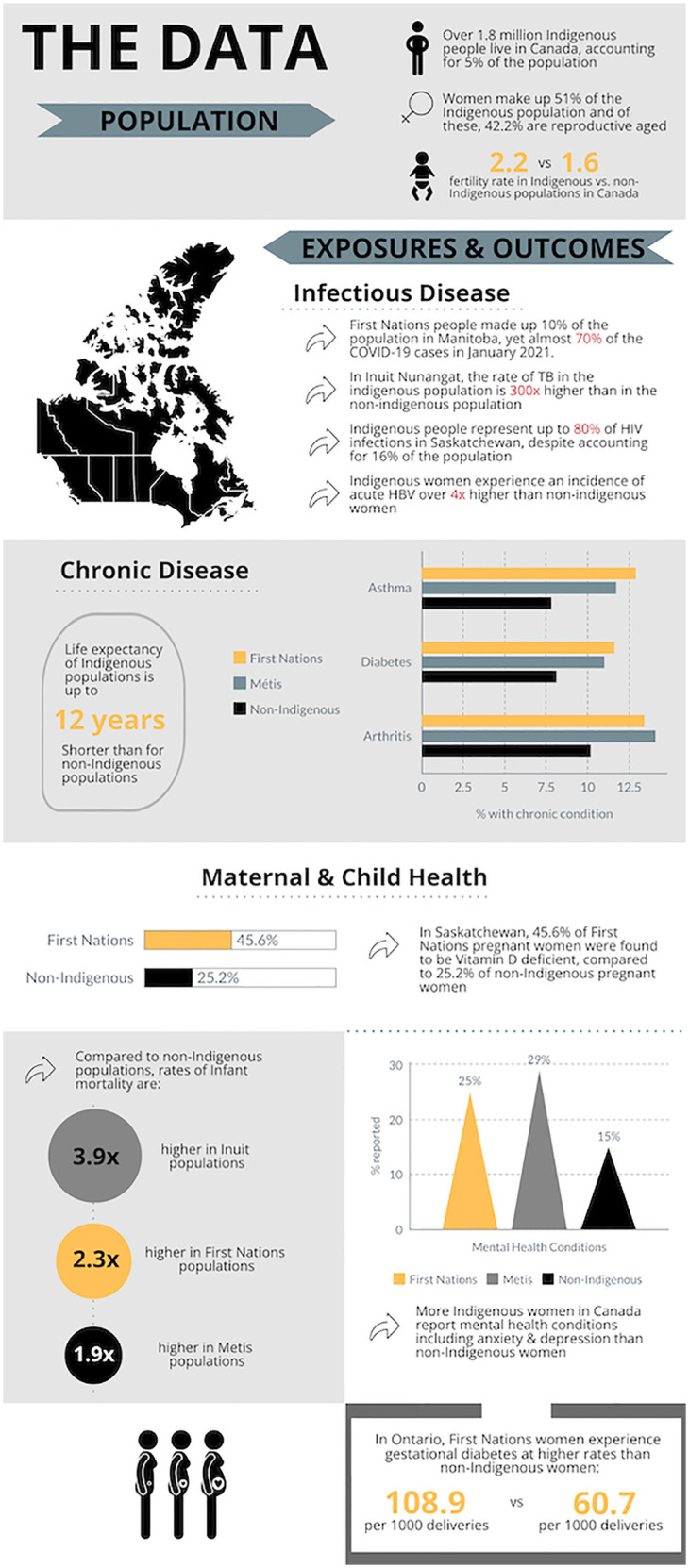
Infographic depicting demographics of the Indigenous population in Canada and exposures and outcomes disproportionately experienced by indigenous populations in Canada, including infectious disease, chronic disease, and maternal-child and women's health. Data Sources: (4, 45–48). Figure created using Piktochart.

Universal, publicly funded healthcare has long been a point of pride for Canada, despite decades of data indicating its universality and accessibility as debatable, at best. The reality is that Canada's healthcare system does not and has not ever served all peoples equally. Inequities in access to and provision of healthcare services are particularly evident in the direct comparison of health outcomes between Indigenous (First Nations, Inuit, and Métis) and non-Indigenous populations in Canada. For example, within one year Indigenous populations were significantly less likely to have seen a physician and significantly more likely to have a chronic condition including arthritis, asthma, diabetes, and obesity (1). In Canada's emergency departments, Indigenous people receive lower acuity triage scores than non-Indigenous people with the same diagnoses and are more likely to leave the ED without being seen (2, 3). Moreover, the life expectancy of Indigenous peoples in Canada is up to 12 years shorter than for non-Indigenous populations (Figure 1) (4).

The overall health of a population can be measured by the health of its most vulnerable—pregnant women and children. In Canada, Indigenous pregnant women experience higher rates of maternal mortality and, according to the Public Health Agency of Canada (PHAC), Indigenous children are two to four times less likely to survive infancy than non-Indigenous children (4). Cause-specific contributors to infant mortality include elevated rates of infection, congenital abnormalities, and conditions related to prematurity in Indigenous infants (5). Most starkly, rates of sudden infant death syndrome (SIDS) are seven times higher (2.0 vs. 0.3 per 1,000 livebirths) for Indigenous infants than non-Indigenous infants (5). Rates of maternal-fetal morbidity are similarly elevated—Indigenous mothers and babies are more likely to have experienced harmful *in utero* exposures (Box 1) (6), reduced access to reproductive age and antenatal healthcare (6, 7), and/or adverse birth outcomes (i.e., preterm birth and large-for-gestational age in Inuit and First Nations communities, respectively) (5) with lifelong consequences for health and development. Contributors to and consequences of these disparities in Indigenous maternal-fetal health are explored below.

BOX 1 **Key definitions, key points, and key resources relevant to this manuscript and Indigenous perinatal health in Canada are outlined here.**

**Key definitions**

• **Harmful *in utero* exposures** refers to perinatal exposures with evidence to support their association with long-term maternal and child health outcomes, including transgenerational epigenetic changes. Examples of such exposures include but are not limited to malnutrition (over- and undernutrition); acute and chronic infections; stress, depression, and/or anxiety; environmental and industrial toxins; substance use (e.g., alcohol, nicotine, cannabis, opioids); comorbid conditions (e.g., hypertension, diabetes, obesity); and adverse birth outcomes [reviewed in (16–18, 43, 44)]

**Key points**

• Indigenous pregnant women in Canada are far more likely to have experienced harmful *in utero* exposures, reduced antenatal care, and adverse birth outcomes than non-Indigenous pregnant women
• These inequities in maternal-child health may be contributing to biological processes (e.g., epigenetic reprogramming) with intergenerational consequences for chronic disease risk in Indigenous populations
• As such, the current state of maternal-child health for Indigenous women in Canada is likely perpetuating the multigenerational cycle of oppression triggered by the process of colonization

**Some prenatal resources for indigenous women in Canada**

• https://www.ontario.ca/page/prenatal-postnatal-and-early-years-support-indigenous-women-children-and-families
• http://www.perinatalservicesbc.ca/health-professionals/professional-resources/indigenous-resources
• https://www.ualberta.ca/wahkohtowin/media-library/data-lists-pdfs/prenatal-services-resources-edmonton1.pdf
• https://www.fnha.ca/what-we-do/maternal-child-and-family-health/maternal-and-child-health
• https://www.snhs.ca/child-youth-health/birthing-centre/
• https://www.fnhssm.com/copy-10-of-new-page
• https://indigenousmidwifery.ca/

It is worth noting that this problem is not unique to Canada. Globally, Indigenous women experience higher rates of maternal morbidity and mortality, receive less antenatal care, and have worse birth outcomes (8–12). In the US, Indigenous women are almost twice as likely to experience severe maternal morbidity or mortality than white women (8). Indigenous mothers in Australia have a maternal mortality incidence more than three times that of non-Indigenous mothers (9). In Namibia, 33% of Indigenous women did not receive antenatal care and 62% had no skilled attendants at delivery, compared to 3% and 11%, nationally (11). In Australia, New Zealand, US, and Canada, the Indigenous to non-Indigenous infant mortality rate ratios are universally elevated, ranging from 1.6 to 4.0 (10). While global Indigenous populations are, of course, highly heterogeneous, many share common experiences of colonization, environmental and land dispossession, and ongoing systemic racism that contribute to worse maternal-child health (12). Neither is this problem unique to Indigenous populations—disparities in access to and outcomes of maternal-child health similarly affect Indigenous and non-Indigenous populations at the intersection of multiple forms of marginalization and oppression (e.g., race/ethnicity, language, newcomer status, education level, rurality, incarceration, socioeconomic status, gender, sex, and sexuality, etc.) (13).

In Canada, the intergenerational effects of complex trauma on Indigenous population health, in the context of residential schools and ongoing colonialist policies, have been well-documented (14, 15). However, the potential for compounding intergenerational, epigenetic effects of adverse *in utero* exposures and birth outcomes on Indigenous population health have not been emphasized. Here, we emphasize this potential and outline important efforts to break the cycle and achieve reproductive justice for Indigenous populations in Canada.

## The link between prenatal health and lifelong health

An immense body of research supports the developmental origins of disease, whereby a child's prenatal and early life exposures directly impact their lifelong health trajectory (16). Many maternal-fetal exposures during pregnancy (e.g., stress, malnutrition, infection) have been associated with long-term cardiovascular, metabolic, immune, and neuropsychiatric morbidities for mother and child, emphasizing the need for comprehensive prenatal care and maternal disease prevention (Box 1) (16). Furthermore, harmful *in utero* exposures and adverse birth outcomes (e.g., preterm birth) have been shown to induce epigenetic reprogramming and shifted risk profiles in the developing baby with lasting, multigenerational consequences for neurodevelopment and chronic disease (17, 18). For example, a child born preterm is at higher risk for hypertension, cardiovascular, and renal disease later in life. Moreover, female children born preterm are at increased risk for preterm delivery of their own children, creating an intergenerational cycle that perpetuates and amplifies the sequelae of preterm birth (18). There is also clinical and preclinical evidence for the transgenerational transmission of metabolic (e.g., elevated adiposity, impaired glucose regulation, BMI) and behavioural traits (e.g., stress hyperreactivity, depressive behaviours) due to parental malnutrition, infection, stress, and toxin exposures (17). Similarly, the intergenerational transmission of complex trauma is hypothesized to have a physiological basis in epigenetic programming (15).

In Canada, all the maternal exposures and birth outcomes, as well as the long-term chronic diseases described above are disproportionately prevalent amongst Indigenous populations (Figure 1) (1). Indigenous women in Canada are more likely than non-Indigenous women to have gestational diabetes and nutritional deficiencies during pregnancy for multifactorial reasons including higher rates of pre-gestational diabetes, lack of culturally appropriate health and nutrition counselling, and food insecurity secondary to social, economic, and geographical inequities (6). Rates of acute and chronic infections including COVID-19, HIV, Hepatitis A and B, HPV, *Chlamydia trachomatis,* and tuberculosis, are higher in Indigenous populations than non-Indigenous populations across Canada (19). This is especially true for more isolated Indigenous communities in the prairies and Northern communities, where multiple socioeconomic determinants of health (e.g., underlying medical conditions, crowded housing, poverty, poor access to healthcare and treatment) intersect to underlie higher infection rates and worse outcomes (19). Resource extraction economies in many Indigenous lands are linked to higher rates of environmental toxin exposure [e.g., mercury, polychlorinated biphenyl (PCB), uranium, pesticides] and gender-based violence during pregnancy (6, 20). Elevated rates of stress, anxiety, and depression reported by Indigenous women during pregnancy are compounded by colonialist maternal health policies including legislatively enforced evacuation from their rural communities to deliver at faraway urban hospitals (6). The residential school and reservation systems created a cycle of economic isolation and reduced employment opportunities for Indigenous peoples, leading to higher unemployment rates, wage gaps, and lower socioeconomic status—well-established social determinants of health and pregnancy outcomes (1, 21). High rates of poor housing conditions and overcrowded households contribute to an increased risk for preterm birth amongst Indigenous peoples (22). The intersectional effects of high-risk determinants of substance use (i.e., history of sexual abuse and/or intergenerational trauma, childhood separation, low socioeconomic status, unstable housing, reproductive injustice, etc.) have also put Indigenous women at disproportionate risk for perinatal substance use (23).

Collectively, these data indicate the possibility for convergence of complex trauma and harmful *in utero* exposures on epigenetic pathways in Indigenous communities, with long-term population health consequences. As such, widespread disparities in maternal-child health faced by Indigenous communities in Canada may be exacerbating the health impacts caused by multigenerational cycles of oppression, trauma, and intersectional marginalization brought on by colonization.

## Antenatal and postnatal care

Reproductive and antenatal healthcare are critical for preventing or mitigating *in utero* exposures and adverse pregnancy outcomes that have intergenerational impacts (16) and Indigenous women access and/or receive reproductive age and antenatal care at lower rates than non-Indigenous women (7). For example, a large population-based study in Canada showed that despite reporting worse health and more chronic diagnoses, 40.7% fewer Inuit women of child-bearing age had access to a regular health care provider and 21.5% more First Nations women of child-bearing age reported unmet mental healthcare needs, relative to non-Indigenous women (7). Amongst pregnant women or new mothers, PHAC reports that 71% of Indigenous vs. 89% of non-Indigenous women have a regular healthcare provider (24).

These disparities are due to a myriad of factors including geographic and economic barriers; the colonization of childbirth and a lack of culturally appropriate care (e.g., the Indian Act, legislative outlawing of Indigenous midwives, dismissal of Indigenous knowledge and agency surrounding maternity and childbirth, Health Canada enforced evacuation); and extraordinary levels of anti-Indigenous racism/stigmatization in the reproductive healthcare system including forced or coercive sterilization and child apprehension (6, 7, 25–27). Canada's long history of child apprehension—residential schools through to the Sixties Scoop and present-day overrepresentation of Indigenous children in child welfare systems—has had direct effects on maternal health, reducing access to antenatal care for subsequent pregnancies in women with a history of child apprehension (26). Beyond the antenatal period, the labor/delivery and neonatal periods including emergency obstetric care, attendance of a skilled provider, and postnatal care are also critical to optimize outcomes. Within the last few years, Pearl Gambler, Sarah Morrison, and Kristy White are three Indigenous women who have made Canadian headlines for speaking out about their abhorrent experiences within the Canadian maternal-child healthcare system (28–30). Each of these women reported mistreatment, neglect, and outright racism during childbirth, ending in the death or injury of their babies.

Indigenous women in Canada face inequities all along the continuum of reproductive care. Here, we have outlined how the repercussions of this do not stop at a single generation. Increasing evidence that perinatal health has lasting, intergenerational impacts for population health suggests that inequities in Canadian maternal-child healthcare are perpetuating the cycle of intergenerational oppression linked to colonization, land dispossession, intersectional marginalization, and systemic anti-Indigenous racism.

## Discussion: where to go from here

The way forward must centre Indigenous voices and perspectives. Several systematic reviews have highlighted the perspectives of Indigenous women on general and perinatal health inequities in Canada, citing service availability, negative experiences with healthcare providers (i.e., racism, discrimination, marginalization), the negative impact of systemic social determinants of health including justice, education, and socioeconomic status, and the impact of deep-rooted colonial ideologies and practices (i.e., medical evacuation) as self-identified factors impacting the health and wellbeing of Indigenous women (31, 32). Steps to achieving reproductive justice and equity that have been suggested by Indigenous researchers, activists, and the Truth and Reconciliation Commission of Canada include implementation of Indigenous community-led models of care, Indigenous representation in research and at decision-making tables, increased representation of Indigenous populations within the healthcare workforce (i.e., midwives, nurses, physicians, etc.), and a systemic shift in healthcare policy, education, and practice to cultural safety, respect for Indigenous knowledge and agency, anti-racism, trauma-informed care, and self-determination for Indigenous peoples (1, 6, 26, 33, 34).

Over the past decade, there has been persistent progress by Indigenous activists and allies in highlighting and addressing the inequities in Indigenous maternal health, mirrored by increasing support and momentum for the reclamation of childbirth by Indigenous communities. These efforts are grounded at the individual level, with increasing empowerment of Indigenous patient and provider voices and leadership in research and programming to inform self-determined strategies and interventions (32, 35–37). At the societal and infrastructural level, we have seen the growth and development of long-standing, sustainable models of Indigenous-owned maternal-child care (e.g., Inuulitsivik midwifery service and education program, Tsi Non:we Ionnakeratstha/Ona:grahsta' Maternal and Child Health Center on the Six Nations of the Grand River Territory) (38, 39); expansion of University- and community-based Indigenous midwifery training programs and resurgence of Indigenous doula training (35, 40, 41); the establishment of nationally recognized Indigenous-led societies (i.e., National Council of Indigenous Midwives); and the adoption of dedicated Indigenous maternal health initiatives by professional associations like the Society of Obstetrics and Gynecology of Canada (SOGC). These efforts have translated into increasing pressure for reform at the government and policy level, which has seen positive steps forward including legislative reforms to the Ontario Midwifery Act, federal Bill C-92 returning jurisdiction over child and family services to Indigenous communities, and the allocation of provincial and federal funding to support Indigenous midwifery training and birthing centers.

Despite this progress, the equity gap has not been closed (33). As outlined above, the data that exist to quantify *in utero* exposures, access to reproductive and antenatal care, and birth outcomes in Indigenous vs. non-Indigenous, Canadian-born populations suggest that there is work yet to be done. There are strong, evidence-based and scalable models for Indigenous-led community programs that work to improve maternal-child health and there are a plethora of frameworks and calls to action that clearly outline solution-oriented steps required to continue moving forward (33, 36, 38, 39, 42). The foundations for a more equitable system have been laid and it is our responsibility to continue building on them.

It is far past time to prioritize the decolonization of Indigenous maternal-child health. Canada's universal healthcare system is not universal, but it can become more equitable with cultural humility and institutional and policy reform informed by those who are affected. We are a new generation of healthcare practitioners who have been exposed and educated more than any generation before us to the realities of healthcare for Indigenous peoples in Canada. We know better and so we must do better.

## Positionality

AMW is a white woman who lives and studies on the traditional territories of the Mississauga and Haudenosaunee nations, and within the lands protected by the “Dish with One Spoon” wampum agreement. She holds a PhD in global maternal-child health from the University of Toronto and is currently a medical student at McMaster University. This publication was a collaborative effort, guided by the insight and teachings of PF, as part of a self-reflective journey in the context of an elective course on Indigenous Health in Canada. It reflects her commitment to addressing maternal health disparities in under-served communities and advocating for culturally competent, equitable healthcare.

PF is an Annishinaabe Scholar from Saugeen-Ojibway territory in Ontario. As the Chair of Indigenous Health for McMaster Undergraduate Medical School Program, she provides education, teaching, and opportunities for all students at McMaster in Indigenous Ways of Knowing, to work towards cultural humility.

## Data Availability

The original contributions presented in the study are included in the article/Supplementary Material, further inquiries can be directed to the corresponding author.
